# DNA损伤修复与肺癌顺铂耐药机制的研究进展

**DOI:** 10.3779/j.issn.1009-3419.2011.12.11

**Published:** 2011-12-20

**Affiliations:** 400038 重庆，第三军医大学西南医院呼吸内科 Department of Respiratory Medicine, Southwest Hospital, Third Military Medical University, Chongqing 400038, China

**Keywords:** 肺肿瘤, 顺铂, 耐药机制, DNA损伤修复, Lung neoplasms, Cisplatin, Resistance mechanism, DNA damage repair

## Abstract

肺癌是目前世界上致死率最高的恶性肿瘤, 化疗是治疗的主要手段之一, 肺癌的化疗是以铂类为基础的联合化疗, 其中, 顺铂是有效并广泛应用的一线药物, 但是由于耐药问题的存在使其疗效不尽如人意。顺铂是一种细胞周期非特异性细胞毒药物, 其主要作用靶点是DNA, 因此DNA损伤修复功能的异常是顺铂耐药的主要机制之一。本文主要综述了与肺癌顺铂耐药相关的DNA损伤修复异常, 包括核苷酸切除修复异常、碱基错配修复异常、DNA双链断裂损伤修复异常及跨损伤修复。

## Abstract (en)

Lung cancer is the most common cause of death from cancer worldwide per year.Platinum-based combination chemotherapy is a main treatment of lung cancer.Cisplatin is adopted widely and used effectively in the first-line chemotherapy.Unfortunately, development of cisplatin resistance is a major obstacle to the success of lung caner.Cisplatin is a cell-cycle-non-specific cytotoxic drugs and its main target is DNA.Thus, defective DNA damage repair is one of the main mechanisms of cisplatin resistance.In this review, we will focus on the defective DNA damage repair in cisplatin resistance of lung cancer including nucleotide excision repair, DNA mismatch repair, DNA double-strand break repair and translesion synthesis.

肺癌是目前世界上发病率及死亡率最高的恶性肿瘤, 研究^[1]^表明, 至少有40%的肺癌患者在确诊时已为晚期, 仅有约25%的患者为Ⅰ期, 而对于晚期及部分中期及术后患者, 化疗是治疗的主要手段之一。尽管对肺癌的化学治疗取得了一定的进展, 但过去25年其5年生存率仍未见明显提高, 约为15%^[2]^。肺癌的化疗是以铂类为基础的联合化疗, 其中, 顺铂(cis-dichlorodiamine platinum, cisplatin, CDDP)是有效并广泛应用的一线药物, 但是由于耐药问题的存在使其疗效不尽如人意。

顺铂是一种细胞周期非特异性细胞毒药物, 含有类似烷化剂的双功能基团, 与细胞内亲核基团结合, 无选择地分布在肿瘤组织中, 其主要作用靶点是DNA。顺铂进入肿瘤细胞后水解为双氯双氨铂, 然后与细胞DNA形成顺铂-DNA加合物, 通过DNA-Pt-DNA结构形成DNA链内交联、链间交联或通过DNA-Pt-蛋白质形成DNA-蛋白交联, 破坏DNA的正常结构, 阻碍DNA的模板作用, 进而抑制DNA的复制和转录, 诱导细胞凋亡^[3, 4]^。因此, DNA损伤修复功能的异常是顺铂耐药的主要机制之一。

DNA损伤修复系统在抗癌过程中起到极为重要的作用, 其功能增强会使肿瘤细胞对化疗药产生拮抗, 使化疗失败^[5]^, 同时受损DNA的异常修复可能会增加患癌症的风险。机体对DNA的损伤修复包括:①直接修复(direct repair, DR):修复O^6^-烷基-鸟嘌呤引起的损伤; ②碱基切除修复(base excision repair, BER):针对氧化还原或烷基化引起的碱基损伤; ③核苷酸切除修复(nucleotide excision repair, NER); ④DNA错配修复(mismatch repair, MMR); ⑤DNA双链断裂损伤修复; ⑥跨损伤修复(translesion synthesis, TLS)。本文主要综述与肺癌顺铂耐药相关的DNA损伤修复异常。

## 1 NER

DNA的损伤修复过程包括损伤识别、切开、切除、修复合成和DNA连接等5个步骤, NER的修复通过切除药物或者紫外线等损伤的DNA而形成的DNA加合物, 以互补链为模板复制并修复损伤DNA来维护基因组的完整性。其中, DNA损伤的识别/切除为限速步骤。

### 1.1 切除修复交叉互补基因1(excision repair cross-complementing gene 1, ERCC1)

ERCC1位于染色体19q13.2, 基因全长15 kb, 编码含297个氨基酸的蛋白质, ERCC1与XPF形成的异二聚体具有5′DNA核酸内切酶活性, 具有损伤识别和切除5′端的双重作用, 在NER中起到限速或调节的重要作用^[6]^, 其活性的高低可反映整个NER修复活性的水平^[7]^。ERCC1过表达可使停滞在G_2_/M期的损伤DNA迅速修复, 尤其是ERCC1能使顺铂诱导的DNA络合物的清除增加, 导致其对顺铂耐药。此外, 研究^[8, 9]^表明, ERCC1-XPF复合物也可以通过同源组合修补DNA双联断裂损伤从而引起肺癌顺铂耐药。

研究^[10, 11]^表明, ERCC1在肺癌组织和细胞系中均存在过表达, 其表达水平以肺鳞癌最高, 而肺腺癌最低。Arora等^[9]^采用RNA干扰的方法降低非小细胞肺癌(non-small cell lung cancer, NSCLC)细胞中ERCC1-XPF的表达, 发现XPF蛋白水平随ERCC1下调而减低, 顺铂诱导的DNA损伤修复能力随XPF、ERCC1、ERCC1-XPF表达的下调而降低, 链内交联引起的DNA双链断裂在XPF-ERCC1缺失时持续存在, 进一步研究发现, ERCC1-XPF复合物抑制的细胞活性减弱, 对顺铂敏感性增加, 且同时抑制XPF和ERCC1较单独抑制XPF或ERCC1细胞毒性更强。在临床研究中也发现, ERCC1的表达水平与肺癌以铂类(主要是顺铂)为基础的化疗临床结果负相关。Wang等^[12]^应用免疫组化方法检测了ERCC1在124例晚期NSCLC的石蜡切片的表达情况, 回顾性地分析了它们对以铂类为基础的化疗患者的化疗有效性及生存期的预测作用。结果显示, ERCC1的阳性表达率为35%(43/124), ERCC1的表达与肿瘤对化疗的反应负相关, 没有表达ERCC1的患者部分缓解率为54%, 而表达该蛋白的患者仅为33%, 具有统计学意义(*P*=0.022), 进一步分析, 缺乏ERCC1表达的患者具有更长的中位生存期(13.4个月*vs* 9.1个月, *P*=0.006)。Li等^[13]^通过对66例晚期NSCLC未治疗前经支气管肺活检标本中ERCC1 mRNA表达水平检测得出结论, ERCC1低表达接受以顺铂为基础的化疗效果较好, 总生存期(overall survival, OS)延长。但是, 对于不予化疗的肺癌患者, ERCC1的阴性表达可能为生存预后较差的指标。Olaussen等^[14]^采用免疫组化方法检测了国际肺癌临床试验入组的761例NSCLC患者手术切除肿瘤组织中的ERCC1的表达情况, 其中ERCC1阳性335例(44%), ERCC1阴性426例(56%)。ERCC1阴性的患者随机接受辅助化疗明显延长生存时间(*P*=0.002);ERCC1阳性患者无论是否接受辅助化疗与否, 生存情况没有统计学差异(*P*=0.400);对于没有接受辅助化疗的患者, ERCC1阳性患者生存时间长于ERCC1阴性患者(*P*=0.009)。因此他们认为:手术完全切除的ERCC1阴性的NSCLC患者更适合基于顺铂的辅助化疗; 对于未予辅助化疗的患者, ERCC1阴性是预示着较短生存期的独立预后因素。以上研究表明, ERCC与肺癌顺铂原发耐药相关, 同时也说明ERCC1在肺癌中具有双重作用, 此与近年认为DNA损伤修复是双刃剑的理论相符。此外, *ERCC1*基因的多态性也能影响肺癌顺铂耐药^[15]^。

### 1.2 核糖核苷酸还原酶M1(ribonucleotide reductase, RRM l)

RRM1是核苷酸还原酶的组成部分, 而核苷酸还原酶是脱氧核苷酸产生的关键酶, 是DNA合成与修补必需酶^[16]^, 也是DNA合成及修复的限速酶, 其基因定位于染色体11p15.5。在NER途径中顺铂加合物导致的核苷酸损害, 所造成的间隙由RRM1提供的DNA合成前体所充填, 执行分裂沟填充的功能。此外RRM1还参与吉西他滨代谢。

目前认为, RRM1是判断NSCLC恶性行为的一个生物学和临床的重要指标。*RRM1*是肿瘤抑制基因^[17]^, 其表达可抑制肿瘤细胞增殖。Bepler等^[18]^研究表明, 在多因素分析中, RRM1表达是预测生存延长的指标, RRM1与PTEN表达增高的患者与表达降低的患者相比较其生存延长, 复发延迟。但在化疗损伤后DNA修补期间, 却发现人类RRM1被上调^[19]^, 但却对化疗的反应性下降。Wang等^[12]^应用免疫组化方法检测了RRM1在124例晚期NSCLC的石蜡切片的表达情况, 回顾性的分析了它们对以铂类为基础的化疗患者的化疗有效性, 结果显示RRM1的阳性表达率为40%(50/124), 且RRM1的表达与肿瘤对化疗的反应负相关, 没有表达RRM1的患者部分缓解率为54%, 而表达该蛋白的患者仅为36%, 具有统计学意义(*P*=0.042)。然而, 尽管RRM1高表达的晚期NSCLC患者接受以吉西他滨+顺铂化疗具有更差的临床结果, 但是接受手术的NSCLC患者, RRM1高表达则具有更长的生存期^[15]^。以上研究说明RRM1可能与顺铂的获得性耐药相关。

## 2 DNA错配修复

MMR是细胞复制后一种修复机制, 该系统的主要功能是修复DNA的碱基错配损伤, 以保持DNA忠实复制, 控制基因突变, 在保持基因的完整性上具有重要作用。目前共发现人类有9种错配修复基因(human mismatch repair genes, hMMR genes), 即*hMLH1*、*hMLH3*、*hMSH2*、*hMSH3*、*hMSH4*、*hMSH5*、*hMSH6*、*hPMS1*和*hPMS2*。其中, *hMLH1*和*hMSH2*的作用最为重要^[3]^。

研究^[20-22]^表明, 在肺癌组织中普遍存在错配修复基因的低表达, 尤其是在吸烟的男性患者中更为突出, 提示DNA错配修复功能的缺陷与肺癌的形成有关。此外, 错配修复基因的表达也与肺癌顺铂耐药相关。Scartozzi等^[23]^应用免疫组化方法检测了93例晚期NSCLC肿瘤标本中hMLH1和hMSH2的表达情况, 所有的患者均接受顺铂或奥沙利铂与吉西他滨的联合化疗, 结果显示, 缺失hMSH2表达的患者, 奥沙利铂+吉西他滨组的化疗应答率为38%, 而顺铂+吉西他滨组完全无效, 提示对hMSH2缺失对顺铂耐药; 最近, Cheng等^[24]^研究报道了*hMSH2*和*hMLH1*单核苷酸多态性与接受以铂类(顺铂或卡铂)为基础化疗的晚期NSCLC患者对化疗反应的关系。一共对96例中国晚期NSCLC患者进行了检测, 取其外周血淋巴细胞通过三维聚丙烯酰胺凝胶脱氧核糖核酸微阵列方法分析hMSH2 gIVS12-6T/C和hMLH1-1151T/A与化疗的关系, 结果发现, hMSH2 gIVS12-6T/C化疗效果更好。吴等^[25]^研究了*hMLH1*基因启动子甲基化与NSCLC细胞顺铂耐药的关系。他们选择A549亲代及顺铂耐药细胞株进行研究, 结果显示, hMLH1 mRNA在A549中的表达高于A549/DDP细胞, A549细胞*hMLH1*为非甲基化状态, A549/DDP细胞为部分甲基化状态, 经5-氮杂-2’-脱氧胞苷(5-Aza-CdR)去甲基作用后, *hMLH1*呈非甲基化状态; A549组、A549/DDP组、经10 μmol/L 5-Aza-CdR干预后的A549/DDP组经顺铂作用后的IC_50_值分别为(4.7±0.7)μmol/L、(30.1±1.8)μmol/L和(6.9±0.6)μmol/L; 5-Aza-CdR干预后的A549/DDP细胞经顺铂作用后, 比单用顺铂抑制A549/DDP细胞的凋亡形态学改变明显, 凋亡小体以及核固缩现象增多, 因此作者认为:*hMLH1*甲基化可能参与了A549/DDP细胞的顺铂耐药过程; 5-Aza-CdR能抑制*hMLH1*甲基化, 恢复hMLH1表达, 并能增强顺铂对A549/DDP细胞增殖抑制和凋亡。

## 3 DNA双联断裂修复

*BRCA1*(breast cancer type 1 susceptibility gene)作为一种抑癌基因, 其表达的蛋白具有重要的生物学功能, 包括细胞周期调控、DNA损伤修复、基因的转录调节、细胞凋亡和泛素化等。BRCA1与MRE1/RAD50/NBS1复合物相互作用, 共同参与DNA双链断裂修复。另有研究还表明BRCA1通过c-JNκ通路参与细胞周期的调控与凋亡^[26]^, 而这些机制都可能与顺铂的耐药相关, 阻止该通路会增加细胞株对顺铂的敏感性^[27]^。

Miquel等^[28]^对接受了吉西他滨/顺铂新辅助化疗的NSCLC手术患者进行研究, 发现BRCA1 mRNA表达水平与患者中位生存期明显相关, BRCA1在NSCLC中高表达患者的生存期明显缩短。尤其是四分位分组时, BRCA1表达最低组的中位生存期还没能得出, 中间两个组的中位生存期为37.9个月, 最高组为12.7个月。Rosell等^[29]^在126例可切除NSCLC患者的9个基因mRNA检测中, 仅有BRCA1 mRNA的表达是Ⅲ期NSCLC独立的预后因素; 40例BRCA1高表达患者中位生存期29个月(22.2个月-35.7个月), 而83例BRCA1低表达者尚未达到, 提示BRCA1高表达是预后差的标志。既往实验^[30]^表明BRCA1过表达可以增加多西紫杉醇的敏感性而对顺铂耐药, Rosell等^[31]^在临床试验中也得出相似的结果, 他们根据123例已发生转移的非鳞癌NSCLC肿瘤标本中BRCA1 mRNA的表达情况选择不同的化疗方案, 低表达者选择顺铂+吉西他滨, 中表达者选择顺铂+多西紫杉醇, 高表达者选择多西紫杉醇, 结果显示2年生存率分别为41.2%、15.6%和0, 提示BRCA1的高水平表达可能会降低以顺铂为基础的联合化疗的2年生存率, 而部分原因可能是由于BRCA1高水平表达者对顺铂耐药, 因此可根据BRCA1的表达水平制定化疗方案。

## 4 TLS

尽管顺铂导致的DNA损伤可以通过核苷酸切除修复及链内交联修复去除, 但仍有一些损伤存在。细胞忽略容忍未修复的DNA损伤的机制就是TLS, 它是由一组专门的DNA聚合酶来执行的, TLS聚合酶可以绕过未修复的DNA损伤继续进行DNA复制。哺乳动物细胞的TLS聚合酶包括polη(POLH)、polι(POLI)、polκ(POLK)、REV1和polζ (REV3和REV7), 每个酶都有专门的作用底物, 对顺铂而言, polη和polζ可以绕过顺铂-GG加合物继续进行DNA复制^[32, 33]^。

TLS耐受顺铂导致损伤的重要性在TLS聚合酶活性缺失的细胞得到明显的体现。对包括肺癌细胞在内的人细胞系中polη活性进行检测, 发现polη活性缺失的细胞对顺铂导致的DNA损伤增加, 对顺铂更为敏感^[34, 35]^。在另一项研究^[36]^中也发现, 比较polη缺失的细胞和用补体致活polη的同样细胞对顺铂、卡铂、奥沙利铂的敏感性, 发现前者更敏感。在临床研究中, Ceppi等^[37]^检测了72例NSCLC患者的石蜡包埋肿瘤标本的polη mRNA的表达情况, 所有患者均接受以铂类为基础化疗, 发现polη的表达水平与患者的生存时间呈负相关, 高表达者生存时间明显短于低表达者(6.9个月*vs* 21.1个月, *P*=0.003), 进一步说明其与肺癌顺铂耐药的相关性。

## 5 结语

肺癌顺铂耐药是由多因素多因子参与的, 任何一种机制都不可能完全解释肺癌顺铂耐药现象的发生, 而且, 一些在临床前研究确认的肺癌顺铂耐药相关因子在临床应用中的意义尚未完全阐明, 有的是刚刚开展。因此, 一方面, 有必要对肺癌顺铂耐药机制继续进行纵深研究; 另一方面, 可以根据目前已经得到的研究结果制定相应的方案克服肺癌顺铂耐药, 如ERCC1、BRCA1的表达水平的不同直接影响NSCLC患者应用顺铂化疗敏感程度及预后, 因此可以根据基因的表达情况来选择性用药, 但目前这些研究还是比较初步的, 虽然已开始了一些前瞻性研究^[38]^, 但大多基于回顾性的研究结果。此外, 顺铂与一些靶向药物的联合应用也是克服其耐药的方法之一, 如VEGF单克隆抗体贝伐珠单抗与顺铂+吉西他滨联合应用治疗非鳞NSCLC, 可提高化疗反应性并延长患者生存时间^[39]^。最后, 可以开发新的铂类药物来克服顺铂的耐药, 这些药物与顺铂部分或完全没有交叉耐药^[40, 41]^。例如, 奥沙利铂由于细胞摄入机制不同(较少依赖于Ctr1)可以导致细胞内药物浓度减少及MMR蛋白不能识别奥沙利铂诱导的DNA损伤从而缺乏交叉耐药性^[42]^; Satraplatin和picoplatin在NSCLC中也显示出较好的临床活性^[41]^; Tang等^[43]^报道picoplatin可以通过提高细胞内的药物浓度对耐顺铂和卡铂的NSCLC仍然具有细胞毒性, 最近的一项Ⅱ期临床实验^[44]^也表明picoplatin对耐其他铂类药物的SCLC显示出较好的临床效果。相信随着肺癌顺铂耐药机制研究的深入长期困扰肺癌化疗的耐药问题必将得以解决。
